# Effects of temperature acclimation on the upper thermal tolerance of two Arctic fishes

**DOI:** 10.1093/conphys/coae001

**Published:** 2024-02-10

**Authors:** Carolyn R Waterbury, Trent M Sutton, Amanda L Kelley, J Andrés López

**Affiliations:** Department of Fisheries, University of Alaska Fairbanks, Fairbanks AK 99705, USA; Department of Fisheries, University of Alaska Fairbanks, Fairbanks AK 99705, USA; Department of Marine Biology, University of Alaska Fairbanks, Fairbanks AK 99705, USA; Department of Fisheries, University of Alaska Fairbanks, Fairbanks AK 99705, USA

**Keywords:** Arctic teleosts, critical thermal maximum, HSP70 expression, thermal plasticity

## Abstract

The thermally dynamic nearshore Beaufort Sea, Alaska, is experiencing climate change-driven temperature increases. Measuring thermal tolerance of broad whitefish (*Coregonus nasus*) and saffron cod (*Eleginus gracilis*), both important species in the Arctic ecosystem, will enhance understanding of species-specific thermal tolerances. The objectives of this study were to determine the extent that acclimating broad whitefish and saffron cod to 5°C and 15°C changed their critical thermal maximum (CT_max_) and HSP70 protein and mRNA expression in brain, muscle and liver tissues. After acclimation to 5°C and 15°C, the species were exposed to a thermal ramping rate of 3.4°C · h^−1^ before quantifying the CT_max_ and HSP70 protein and transcript concentrations. Broad whitefish and saffron cod acclimated to 15°C had a significantly higher mean CT_max_ (27.3°C and 25.9°C, respectively) than 5°C-acclimated fish (23.7°C and 23.2°C, respectively), which is consistent with trends in CT_max_ between higher and lower acclimation temperatures. There were species-specific differences in thermal tolerance with 15°C-acclimated broad whitefish having higher CT_max_ and HSP70 protein concentrations in liver and muscle tissues than saffron cod at both acclimation temperatures. Tissue-specific differences were quantified, with brain and muscle tissues having the highest and lowest HSP70 protein concentrations, respectively, for both species and acclimation temperatures. The differences in broad whitefish CT_max_ between the two acclimation temperatures could be explained with brain and liver tissues from 15°C acclimation having higher HSP70a-201 and HSP70b-201 transcript concentrations than control fish that remained in lab-acclimation conditions of 8°C. The shift in CT_max_ and HSP70 protein and paralogous transcripts demonstrate the physiological plasticity that both species possess in responding to two different acclimation temperatures. This response is imperative to understand as aquatic temperatures continue to elevate.

## Teaser

Acclimating broad whitefish (*Coregonus nasus*) and saffron cod (*Eleginus gracilis*) to 5#x00B0;C and 15#x00B0;C resulted in a shift in temperature tolerances through changes in CT_max_ and HSP70 protein and mRNA expression. These responses indicate that these two species have the potential to acclimate to temperature changes associated with climate change.

## Introduction

Broad whitefish (Iñupiaq name—Aanaakłiq) and saffron cod (Iñupiaq name—uugaq) are two species of Arctic fishes that co-occur in the nearshore Beaufort Sea, Alaska, during the ice-free period (Wolotira, 1985; Fechhelm *et al.,* 1992; Griffiths *et al.,* 1992; Tallman and Reist, 1997; Mueter *et al.,* 2016). In this region, both species occupy an important intermediate trophic level connecting primary producers and higher trophic consumers (Frost and Lowry, 1981; Tallman and Reist, 1997; Reusser *et al.,* 2016). Additionally, broad whitefish is an important subsistence resource for Indigenous coastal Alaskan communities (Fechhelm *et al.,* 1992; Tallman and Reist, 1997). Anthropogenic-driven climate change is impacting the nearshore area and has been linked to an increase in aquatic temperatures, with some reports indicating an increase four times faster than any other region (Rantanen *et al.,* 2022). Additionally, this area is inherently thermally dynamic and can experience unstable temperatures due to wind-driven currents and river discharge (Mccain *et al.,* 2014; Hamman *et al.,* 2021). Environmental thermal variability is one of the most important abiotic factors regulating fish abundance and distribution as poikilothermic organisms (Reist *et al.,* 2006; Somero, 2010; Zhang and Kieffer, 2014; Khalsa *et al.,* 2021). Some aspects of these species’ thermal tolerances have been studied (Bilyk and Sformo, 2021), but understanding how broad whitefish and saffron cod may respond to increasing environmental temperatures changes at an organismal and molecular level will aid in predicting the impact and response of these species as climate change continues in the nearshore Beaufort Sea.

There are a variety of biological processes that work in concert to produce an organism’s thermal tolerance (Yamashita *et al.,* 2010). One important biological process is the induced expression of the molecular chaperones known as inducible heat shock proteins (HSP), which are upregulated due to a stressor, such as elevated temperature, to maintain cellular homeostasis (Dietz and Somero, 1993; White *et al.,* 1994; Iwama *et al.,* 1998; Somero, 2010). The 70-kDa HSP (HSP70) is the most highly conserved induced HSP across teleost species, and there are several HSP70 transcripts that can have unique species and tissue-specific expression patterns (Lindquist, 1986; White *et al.,* 1994; Iwama *et al.,* 1998; Feder and Hofmann, 1999; Santoro, 1999; Basu *et al.,* 2002; Yamashita *et al.,* 2010). Additionally, the production of HSP can change depending on the thermal environment (Hofmann, 1999).

Organisms can shift their HSP production in different thermal regimes, which can result in a phenotypically plastic upper thermal tolerance threshold, allowing organisms to persist across a range of temperatures (Dietz and Somero, 1992; Basu *et al.,* 2002; Somero, 2010). For example, seasonal temperature changes have resulted in summer-acclimated fish having higher levels of constitutive HSP and higher induction temperatures for the inducible cognate versus winter-acclimated populations (Dietz and Somero, 1992; Dietz, 1994; Hofmann, 1999; Currie *et al.,* 2000). The critical thermal maximum (CT_max_) is one such plastic physiological trait commonly measured as an indicator of an organism’s upper thermal tolerance threshold, and it is measured as the highest sustained temperature resulting in physiological impairment, such as loss of equilibrium, but preceding mortality (Beitinger *et al.,* 2000; Zhang and Kieffer, 2014). Measuring CT_max_, the underlying HSP70 gene expression and protein synthesis, and how these parameters change in differing thermal conditions will improve understanding of overall upper thermal threshold and the extent that it can shift in response to environmental changes (Basu *et al.,* 2002).

The objectives of this study were to: (1) determine the CT_max_ in broad whitefish and saffron cod at two acclimation temperatures; (2) quantify HSP70 protein concentration in brain, liver and muscle tissues to determine if there are differences among tissue types, acclimation temperatures and species; and (3) measure HSP70 transcript abundance in broad whitefish liver and muscle tissues to test for significant differences between tissue types and acclimation temperatures. This information could aid future predictions in the responses and distribution of these two fishes in a warming climate regime (Reist *et al.,* 2006; Somero, 2010).

## Materials and Methods

### Sample collection

All fish sampling, transport and laboratory experiments were conducted in accordance with the Alaska Department of Fish and Game Aquatic Resource Permit (ADF&G; numbers CF-20-021 and CF-21-009) and the University of Alaska Fairbanks Institutional Animal Care and Use Committee (IACUC) protocol (protocol numbers 1054743, 1615559 and 197441). Paired fyke nets (1.8 × 1.7 m, with 12.77-mm stretch mesh netting) were deployed 60 m from shore with a lead net and two blocking nets (60 × 1.8 m and 15 × 1.8 m, 25-mm stretch mesh; Supplementary Fig. S1). Salinity and temperature were measured daily using a handheld probe (YSI® Pro20i; YSI Inc., Yellow Springs, Ohio). Broad whitefish (*n* = 17) and saffron cod (*n* = 35) were sampled in 2020 and 2021, respectively (Supplementary Table S2). Fish were placed in polyethylene bags (15–20 individuals per bag) with Beaufort Sea water before being shipped by air to the University of Alaska Fairbanks (UAF). At UAF, fish were placed in a recirculating rearing tank system maintained at 8°C to lab-acclimate for 6 weeks. Broad whitefish were kept at a salinity of 3.5 ppt while saffron cod were held at a salinity of 9.0 ppt throughout the duration of acclimation and experimentation periods. These salinities were chosen based on the conditions these species have been found in (Reusser *et al.,* 2016). Both species were fed 0.5 g per fish of frozen blood worms (*Glycera* spp*.*) daily.

### Thermal ramping experiment and critical thermal maximum determination

Broad whitefish were divided in to two experimental tanks set to 5°C or 15°C while saffron cod were divided in to two experimental tanks per acclimation temperature (four tanks total) (76.2 × 45.72 × 30.48 cm, 110 l), and both species were allowed to acclimate for a week (Dyer *et al.,* 1991; Lutterschmidt and Hutchison, 1997). These temperatures were the mean minimum and maximum temperatures experienced from 30 June to 22 August 2019 in the nearshore Beaufort Sea (Gatt *et al.,* 2019). Water temperature in the experimental tanks was monitored daily and maintained within 1.0°C of the target temperature. Each tank was fitted with an aquarium air pump (Tetra, Blacksburg, Virginia) and two air stones to maintain oxygen levels. Daily feeding was carried out as described previously, and water changes were conducted daily in the 5°C-acclimation tanks and weekly in the 15°C-acclimation tanks to maintain balanced water chemistry.

At the end of the 1-week acclimation period, fish were placed in individual plastic containers fitted with an air stone and aerator that were placed in a water bath matching the respective acclimation temperature. Thermal ramping was carried out using an 800-W titanium heater fitted in each water bath (Finnex, Chicago, Illinois). The ramping rate was set to 3.4°C · h^−1^, which was the most rapid water temperature rate of change observed in the Beaufort Sea nearshore area during the 2019 ice-free period (Gatt *et al.,* 2019). Temperature was monitored and adjusted to ensure there was a linear increase in water temperature matching the ramping rate (Supplementary Fig. S2). Individual fish were continually monitored until they demonstrated a loss of equilibrium (LOE), which occurred once the fish turned over and could not right itself after 5 seconds. The temperature at which this occurred was recorded as the CT_max_ end-point (Becker and Genoway, 1979; Saravia *et al.*, 2021). Fish were then immediately euthanized with an overdose (100 mg/l) of MS-222 before fork length (in millimeters) and wet weight (in grams) were measured, and a 1-cm^2^ section of pectoral muscle and liver tissue and the entirety of brain tissue were removed and placed into individual cryovials, flash-frozen in liquid nitrogen and stored at −80°C for future analysis. All dissecting instruments were sterilized with 70% molecular-grade ethanol and wiped clean between each tissue collection.

### HSP70 protein concentration quantification

Total protein was extracted from each tissue sample using a homogenization buffer (Supplementary Table S1), and the protein concentration was quantified using the Pierce™ Coomassie (Bradford) Protein Assay Kit (ThermoFisher Scientific, Waltham, Massachusetts). A western blot assay was carried out following the methods in Kelley *et al.* (2013). Briefly, 10 μg of total protein from each sample was separated by electrophoresis on 10% polyacrylamide gels. Proteins were transferred to nitrocellulose membranes and incubated in monoclonal mouse anti-HSP70 antibody (1:1000) and a donkey anti-Mouse IgG (H + L) secondary antibody (1:10000; Supplementary Information 1). Nitrocellulose membranes were exposed to chemiluminescence (SuperSignal reagent; Pierce, Rockford, Illinois) and imaged using the Amerhsam Imager chemiluminescent setting (GE Healthcare, Chicago, Illinois; Supplementary Fig. S3). HSP70 protein concentrations were quantified using ImageJ (v. 1.8.0_172) following the protocol outlined by ImageJ (Abràmoff *et al.,* 2004) and Gassmann *et al.* (2009) to calculate the optical density (OD) unit. An internal standard (broad whitefish fish #5 brain tissue) was used to normalize measurements between blots.

### RNA-seq and HSP70 transcript quantification

Broad whitefish that remained in lab-acclimation conditions at 8°C were used as control samples for mRNA quantification. The transcriptomes from liver (${n}_{5{}^{\circ}\mathrm{C}}$= 6; ${n}_{15{}^{\circ}\mathrm{C}}$ = 6; ${n}_{control}$ = 4) and muscle (${n}_{5{}^{\circ}\mathrm{C}}$ = 5; ${n}_{15{}^{\circ}\mathrm{C}}$ = 6; ${n}_{control}$ = 4) were obtained from Poly(A) selected sequencing libraries using 2x150 chemistry on a Illumina instrument. Transcript abundance was determined using the functions implemented in Salmon (v. 1.6.0, github.com/COMBINE-lab/salmon). Rainbow trout (*Oncorhynchus mykiss*) gene annotation dataset (downloaded from Ensembl v. 105) was used as the reference transcriptome. The quantification values were normalized using transcript per million (TPM) method (Li and Dewey, 2011). The TPM values in two protein-coding paralogous HSP70 transcripts (HSP70a-201 and HSP70b-201) were analysed for differential gene expression between the control and temperature-treated samples (Ojima *et al.,* 2005a).

**Table 1:** The mean CT_max_ and HSP70 protein concentrations for broad whitefish and saffron cod at both acclimation temperatures. Additionally, the HSP70a-201 and HSP70b-201 abundances for broad whitefish are provided

**Acclimation temperature**	**CT**_**max**_ **(** ^**o**^ **C)**	**Tissue**	**HSP70 concentration (OD unit)**	**mRNA expression (TPM)**
*Broad whitefish* (93–148 mm, 8.6–32.0 g)				
5^o^C (*n* = 8)	23.7 ± 1.36	Brain	0.95 ± 0.06	NA
		Liver	0.80 ± 0.26	135 ± 116 (HSP70a-201) 238 ± 197 (HSP70b-201)
		Muscle	0.47 ± 0.14	106 ± 29.2 (HSP70a-201) 210 ± 57.9 (HSP70b-201)
15^o^C (*n* = 9)	27.3 ± 1.06	Brain	0.87 ± 0.10	NA
		Liver	0.61 ± 0.07	443 ± 389 (HSP70a-201) 844 ± 734 (HSP70b-201)
		Muscle	0.32 ± 0.19	760 ± 388 (HSP70a-201) 1657 ± 953 (HSP70b-201)
Control (*n* = 4)	NA	Liver	NA	3.97 ± 2.39 (HSP70a-201) 8.34 ± 2.68 (HSP70b-201)
		Muscle	NA	42 ± 62.7 (HSP70a-201) 92.2 ± 136 (HSP70b-201)
*Saffron cod* (121–170 mm, 8.0–26.1 g)				
5^o^C (*n* = 15)	23.2 ± 0.79	Brain	0.91 ± 0.10	NA
		Liver	0.21 ± 0.09	
		Muscle	0.04 ± 0.04	
15^o^C (*n* = 16)	25.9 ± 0.66	Brain	0.93 ± 0.16	
		Liver	0.07 ± 0.06	
		Muscle	0.03 ± 0.02	

### Data analysis

All statistical analyses were conducted in R (v. 4.2.1; R core team, 2022; tidyr, tidyselect, dplyr, lubridate, ggplot2, here, MASS, ggsignif, rstatix, lmtest, ggpubr, FSA, outliers; Ahlmann-Eltze and Patil, 2021; Grolemund and Wickham, 2011; Henry and Wickham, 2022; Kassambara, 2023a; Kassambara, 2023b; Komsta, 2022; Müller, 2020; Ogle *et al.,* 2023; Venables and Ripley, 2002; Wickham *et al.,* 2019; Zeileis and Hothorn, 2002). A Grubb’s test was used to identify outliers in the CT_max_ and HSP70 protein concentrations; there were too few data points in the mRNA expression dataset to remove outliers. If a high or low outlier was identified with a *P*-value of ≤0.05, the data value was removed. Data from both species and acclimation temperatures was kept together and a linear regression model was used to determine if fish weight, length and the interaction of these two variables correlated with CT_max._ The datasets were then separated by species and treatment group, but the CT_max_, HSP70 protein concentration and HSP70 mRNA expression datasets failed to meet the normality assumption for parametric tests. As a result, non-parametric statistical analyses were used with an $\alpha$ = 0.05 and Bonferroni-corrected *P*-values. A Wilcoxon rank-sum test was used to compare the CT_max_ values between each acclimation temperature and between species at the same acclimation temperature. The acclimation response ratio (ARR), a measurement used to compare the change in thermal tolerance threshold between species, was calculated as: $\frac{\Delta {CT}_{max}}{\Delta Acclimation\ Temperature}$ (Kelley, 2014). For the HSP70 protein expression, a Wilcoxon rank-sum test was used to compare means of the HSP70 protein concentrations between the acclimation temperatures in the three tissue samples and between species at the same acclimation temperature. A Kruskal–Wallis test was used to determine any difference in protein expression based on tissue type for each species, which was followed by a *post hoc* Dunn’s test to determine which pairwise comparisons were significantly different. A Wilcoxon rank-sum test was used to compare the TPM values between muscle and liver tissue samples at the same acclimation temperature. A Kruskal–Wallis test was used to determine if acclimation temperature influenced mRNA expression, and a *post hoc* Dunn’s test was used to determine which pairwise acclimation temperatures were different.

## Results

### Measurement of critical thermal maximum

**Figure 1:**
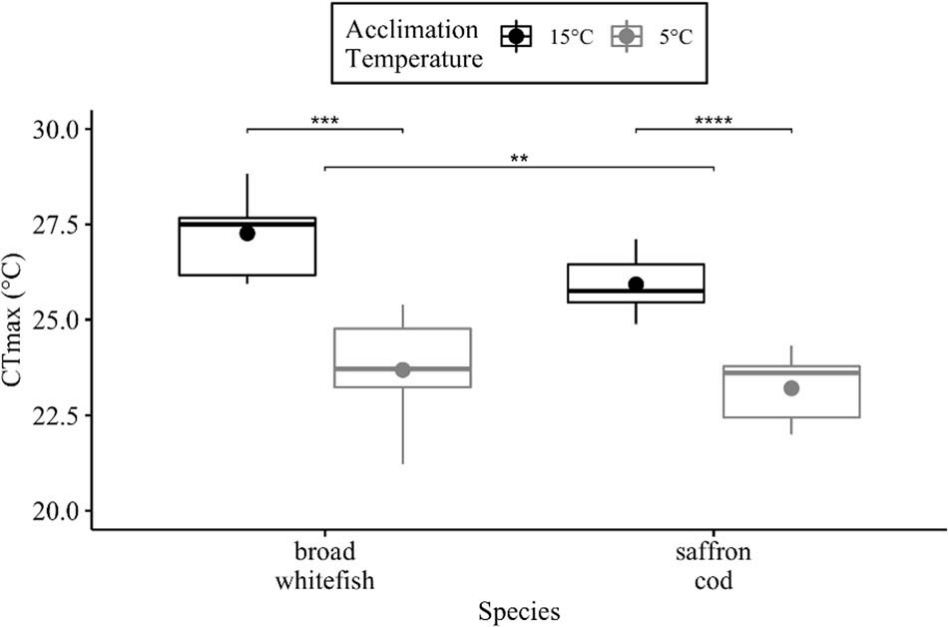
The CT_max_ (°C) for broad whitefish and saffron cod at the 5°C and 15°C acclimation temperatures. The median (line) and mean (dot) values are reported. Significant differences are denoted by ^*^P < 0.05, ^**^P < 0.01, ^***^P < 0.001 and ^****^P < 1 × 10^−4^ from a Wilcoxon rank-sum test.

There was no effect of fish weight (*P* = 0.672), length (*P* = 0.908) or the interaction of weight × length (*P* = 0.633) on the mean CT_max_. For the linear model between acclimation temperature (T_a_) and CT_max_, there was a positive relationship for broad whitefish and saffron cod in addition to broad whitefish having a higher slope than saffron cod (Table 1). The acclimation response ratio for broad whitefish was 0.3895 and 0.2890 for saffron cod. Mean CT_max_ in 15°C-acclimated fish was higher than the group acclimated to 5°C by 3.6°C in broad whitefish (W = 72; *P* < 0.001) and by 2.7°C in saffron cod (W = 224; *P* < 0.0001; Fig. 1; Table 1; Supplementary Table S3). The mean CT_max_ in 15°C-acclimated fish was 1.4°C higher for broad whitefish than saffron cod (W = 110; *P* < 0.01; Fig. 1; Table 1; Supplementary Table S3), but there was no difference between the two species when acclimated to 5°C (W = 81; *P* > 0.05).

**Figure 2:**
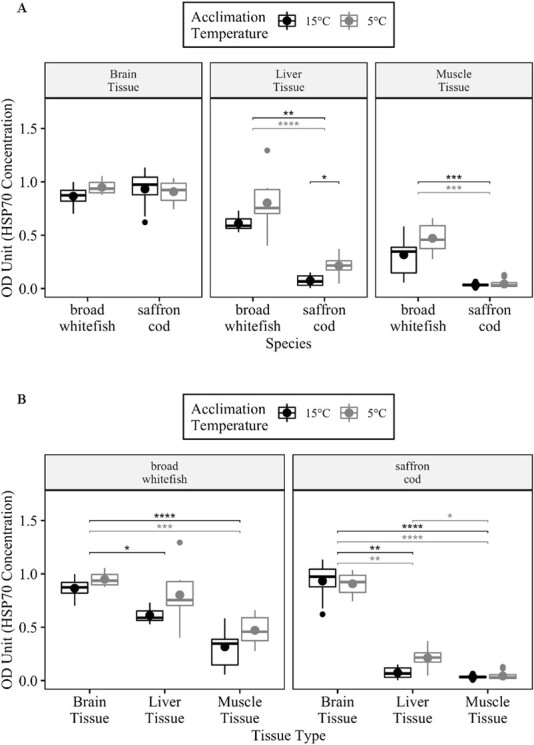
**a.** Comparisons of 70-kDa heat shock protein (HSP70) concentrations (OD unit) between acclimation temperatures 5°C and 15°C and between broad whitefish and saffron cod. The median (line) and mean (dot) values are reported. Significant differences are denoted by ^*^P < 0.05, ^**^P < 0.01, ^***^P < 0.001 and ^****^P < 1 × 10^−4^ from a Wilcoxon rank-sum test. **b.** Comparisons of HSP70 protein concentrations (OD unit) between brain, liver and muscle samples in broad whitefish and saffron cod and between acclimation temperatures 5°C and 15°C. The median (line) and mean (dot) values are reported. Significant differences are denoted by ^*^P < 0.05, ^**^P < 0.01, ^***^P < 0.001 and ^****^P < 1 × 10^−4^ from a Kruskal-Wallis test followed by a Dunn’s *post hoc* test.

**Figure 3:**
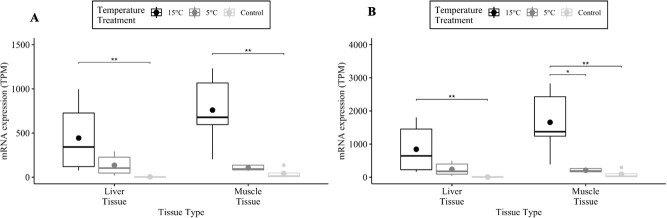
The mRNA HSP70 TPM between broad whitefish liver and muscle tissue samples in addition to between the acclimation temperatures 5°C and 15°C and the control group. The control group were broad whitefish samples that were left in lab-acclimation conditions at 8°C. The image on the left is a result from transcript A, and on the right is transcript B. The median (line) and mean (dot) values are reported. Significant differences are denoted by ^*^P < 0.05, ^**^P < 0.01, ^***^P < 0.001 and ^****^P < 1 × 10^−4^ from a Kruskal-Wallis test followed by a Dunn’s *post hoc* test.

### HSP70 protein synthesis during CT_max_ assay

The antibody used in this study recognizes both the constitutive and inducible HSP70 protein, and thus produces a measure of total HSP 70-kDa protein abundance (Dietz and Somero, 1993; Yoo and Janz, 2003). Under the experimental conditions, any measured changes in HSP70 abundance are expected to be a result of changes in the inducible HSP70 levels (Li *et al.,* 2007). There were no differences in the mean HSP70 protein concentration (hereafter referred to as just protein) between the two acclimation temperatures for the same tissue type (W_liver_ = 12.5; W_muscle_ = 16.0; W_brain_ = 17.0; *P* > 0.05; Table 1; Fig. 2a; Supplementary Table S4). However, tissue type did influence the mean protein concentration (${\chi}_{5{}^{\circ}C}^2$ = 14.6; ${P}_{5{}^{\circ}C}$ < 0.0001; ${\chi}_{15{}^{\circ}C}^2$ = 21.0, ${P}_{15{}^{\circ}\mathrm{C}}$ < 0.0001; Supplementary Table S5). Brain tissue at the 15°C acclimation temperature had a higher mean protein concentration by 43% compared to liver and by almost 3 times compared to muscle (*t*_brain^*^liver_ = −2.5; *P*_brain^*^liver_ < 0.05; *t*_brain^*^muscle_ = −4.6, *P*_brain^*^muscle_ < 0.0001; Table 1; Fig. 2b). However, 5°C-acclimated brain tissue only had a higher mean protein concentration compared to the muscle samples by 102% (*t*_brain^*^muscle_ = −3.8; *P*_brain^*^muscle_ < 0.001; Table 1; Fig. 2b).

The 5°C-acclimated saffron cod liver tissue had three times the protein concentration than the 15°C-acclimated samples (W_liver_ = 8.5; *P* < 0.05; Table 1; Supplementary Table S4), but there was no difference in mean protein concentration in the other two tissues (W_muscle_ = 118.5; W_brain_ = 148; *P* > 0.05; Table 1; Fig. 2a; Supplementary Table S4). Tissue type had an effect on mean protein concentration values (${\chi}_{5{}^{\circ}C}^2$ = 38.5; ${P}_{5{}^{\circ}\mathrm{C}}$ < 0.0001; ${\chi}_{15{}^{\circ}C}^2$ = 26.9, ${P}_{15{}^{\circ}C}$ < 0.0001; Table 1; Supplementary Table S5), with brain tissues having 13.3 and 31 times higher mean protein concentration than liver and muscle at acclimation temperature 15°C (*t*_brain^*^liver_ = −3.1, *P*_brain^*^liver_ < 0.01; *t*_brain^*^muscle_ = −5.1, *P*_brain^*^muscle_ < 0.0001; Table 1; Fig. 2b) and 4.3 and 22.8 times higher protein concentration in liver and muscle at acclimation temperature 5°C (*t*_brain^*^liver_ = −3.2, *P*_brain^*^liver_ < 0.01; *t*_brain^*^muscle_ = −6.2, *P*_brain^*^muscle_ < 0.0001; Table 1). The mean protein concentration in 5°C-acclimated saffron cod liver tissue was five times higher than in muscle (*t*_liver^*^muscle_ = −2.8, *P*_liver^*^muscle_ < 0.05; Table 1; Fig. 2b). Broad whitefish had a higher mean protein concentration than saffron cod by 10.7 times at 15°C and 11.8 times at 5°C in the muscle tissue (${\mathrm{W}}_{15{}^{\circ}C}$ = 133; ${P}_{15{}^{\circ}C}$ < 0.001; ${\mathrm{W}}_{5{}^{\circ}C}$ = 128, ${P}_{5{}^{\circ}C}$ < 0.001; Table 1; Supplementary Table S4), and by 8.7 times at 15°C and 3.8 times at 5°C in liver tissue (${\mathrm{W}}_{15{}^{\circ}C}$ = 63; ${P}_{15{}^{\circ}C}$ < 0.01; ${\mathrm{W}}_{5{}^{\circ}C}$ = 112, ${P}_{5{}^{\circ}C}$ < 0.0001; Table 1; Fig. 2a; Supplementary Table S4).

### HSP70 mRNA abundance

Acclimation temperature had an effect on mean TPM values in liver (${\chi}_A^2$ = 9.9; *P*_a_ < 0.01; ${\chi}_B^2$ = 9.9; *P*_B_ < 0.01; Table 1) and muscle (${\chi}_A^2$ = 11.2; *P*_a_ < 0.01; ${\chi}_B^2$ = 10.7; *P*_B_ < 0.01; Table 1; Supplementary Table S7) tissues. Mean liver and muscle TPM values were 111.6 times higher and 18.1 times higher, respectively, at the 15°C acclimation temperature versus the control group in HSP70a-201 (*t*_liver_ = −3.2, *P*_liver_ < 0.01; *t*_muscle_ = −3.2; *P*_muscle_ < 0.01; Table 1). Mean liver and muscle TPM values were 101.2 times higher and 17.8 times higher, respectively, at the 15°C acclimation temperature versus the control group in HSP70b-201 (*t*_liver_ = −3.2; *P*_liver_ < 0.01; *t*_muscle_ = −3.0; *P*_muscle_ < 0.01; Table 1; Fig. 3). The HSP70b-201 transcript had 7.9 times the mean TPM when acclimated to 15°C versus 5°C in muscle (T = −2.4; *P* < 0.05; Table 1; Fig. 3) tissue. There were no significant differences in mean TPM values between the two tissue types at the same acclimation temperature in HSP70a-201 (${\mathrm{W}}_{15{}^{\circ}C}$ = 9; ${\mathrm{W}}_{5{}^{\circ}C}$ = 13; ${\mathrm{W}}_{control}$ = 2; *P* > 0.05; Table 1; Supplementary Table S6) and HSP70b-201 (${\mathrm{W}}_{15{}^{\circ}C}$ = 9; ${\mathrm{W}}_{5{}^{\circ}C}$ = 13; ${\mathrm{W}}_{control}$ = 0.0; *P* > 0.05; Table 1; Supplementary Table S6).

## Discussion

### Comparison of mean CT_max_ temperatures

The increase in mean CT_max_ as acclimation temperatures increased indicated that both species were successful in shifting their upper thermal tolerance. Between the two species, broad whitefish had a higher acclimation response ratio and CT_max_ at 15°C acclimation than saffron cod, indicating that it exhibits a greater degree of thermal plasticity (Kelley, 2014; Semsar-kazerouni and Verberk, 2018). We expected that broad whitefish would have a lower thermal tolerance and smaller acclimation response ratio than saffron cod since saffron cod has a broader geographic range and experiences higher environmental temperatures than broad whitefish (Fechhelm *et al.,* 1992; Reusser *et al.,* 2016). The CT_max_ and thermal ranges of these species were also compared to data of other fishes from different studies.

It should be noted that the ramping rates used in each study differed with this study, which could impact the CT_max_ data. However, the relevant thermal tolerance information is still important to compare to better understand species-specific differences. The CT_max_ for 5°C- and 15°C-acclimated broad whitefish was lower than reported for most like-acclimated salmonids, and the 5°C-acclimated CT_max_ was within 0.4°C of a CT_max_ previously reported for 9°C-acclimated broad whitefish (Table 2; Bilyk and Sformo, 2021). The 5°C-acclimated saffron cod had a CT_max_ that was higher than reported for 6.5°C-acclimated Arctic cod (*Boreogadus saida*), but the CT_max_ at both acclimation temperatures was lower than other fishes (Table 2; Bilyk and Sformo, 2021). In addition to the CT_max_, there were differences in the thermal ranges (the slope of the CT_max_ equation; CT_max_ = M^*^Temperature_Acclimation_ + B) between these and other species (Table 2). Broad whitefish had a broader thermal range than other salmonids, which may be necessary to tolerate the thermally dynamic nearshore environment of the Beaufort Sea (Table 2; Nati *et al.,* 2021). Conversely, saffron cod had a narrower thermal range than most other comparable fishes, which suggests that this species tolerates a smaller range of temperatures (Table 2). The lower CT_max_ and differences in thermal ranges for broad whitefish and saffron cod compared to the other fishes indicated species-specific thermal tolerances (Table 2; Nati *et al.,* 2021).

**Table 2:** A comparison of different mean CT_max_ and the equations between CT_max_ and acclimation temperature (T_a_) from other teleost studies

**Species name**	**Acclimation temperature (°C)**	**Thermal ramping rate (°C · h** ^**−1**^ **)**	**CT**_**max**_ **temperature ± SD (°C)**	**CT**_**max**_ **equation**	**Reference**
*C. nasus* (broad whitefish)	5	3.4	23.7 ± 1.36	^3^CT_max_ = 0.390^*^T_a_ + 21.18393, R^2^ = 0.6916, *P* < 0.0001	This study
	15	3.4	27.3 ± 1.06		
*E. gracilis* (saffron cod)	5	3.4	23.2 ± 0.786	^3^CT_max_ = 0.289^*^T_a_ + 21.6273, R^2^ = 0.7928, *P* < 0.0001	This study
	15	3.4	25.9 ± 0.655		
*C. nasus* (broad whitefish)	9	18.0	23.3 ± 0.84	NA	(Bilyk and Sformo, 2021)
*B. saida* (Arctic cod)	0.5	3.0	14.9^4^	CT_max_ = 0.43^*^T_a_ + 14.24, R^2^ = 0.74, *P* < 0.0001	(Drost *et al.,* 2016)
	3.5	3.0	15.5^4^		
	6.5	3.0	17.1^4^		
*Coregonus clupeaformis* (lake whitefish)	6	12.0	23.9 ± 0.15	^1^CT_max_ = 0.175^*^T_a_ + 22.867	(Manzon *et al.,* 2022)
	12	12.0	25.0 ± 0.33		
	18	12.0	26.0 ± 0.58		
*Acipenser brevirostrum* (shortnose sturgeon)	10	6.0	27.6 ± 0.35	CT_max_ = 0.52^*^T_a_ + 22.87, R^2^ = 0.803, *P* < 0.001	(Zhang and Kieffer, 2014)
	15	6.0	31.5 ± 0.91		
	20	6.0	32.8 ± 1.17		
*O. mykiss* (rainbow trout)	10	18.0	28.0 ± 0.36	CT_max_ = 0.18^*^T_a_ + 26.23, R^2^ = 0.975, *P* < 0.0001	(Currie *et al.,* 1998)
	15	18.0	29.1 ± 0.27		
	20	18.0	29.8 ± 0.36		
*Oncorhynchus kisutch* (coho salmon)	5	6.0	24.84^4^	NA	(Becker and Genoway, 1979)
	5	18.0	25.32^4^		
	15	6.0	28.13^4^		
	15	18.0	28.70^4^		
*Oncorhynchus clarkii* (cutthroat trout)	10	24.0	27.63 ± 0.08	^2^CT_max_ = 0.255^*^T_a_ + 25.5	(Heath, 1963; Beitinger *et al.,* 2000)
	15	24.0	29.06 ± 0.05		
	20	24.0	29.88 ± 0.09		

^1^= calculated by this researcher based on the paper’s data.

^2^ = no R^2^ or *P*-value was reported for the equation.

^3^= only two acclimation temperatures were used to make the equation.

^4^ = no standard deviation values were reported.

### HSP70 protein synthesis during CT_max_ assay

The few significant differences between HSP70 protein concentration and acclimation temperatures were contrary to other studies that have reported enhanced *in vivo* and *in vitro* protein synthesis at higher acclimation temperatures (Dietz, 1994; Iwama *et al.,* 1999; Dalvi *et al.,* 2012). The observed tissue-specific HSP70 protein concentrations could be explained by underlying differences in the heat shock response between the tissue types, including differences in the constitutive HSP levels, different induction temperatures for the inducible HSP and variations in protein denaturation and proteolysis of damaged proteins (Dietz and Somero, 1993; Smith *et al.,* 1999). Additionally, tissue-specific protein expression patterns have been reported in other teleosts, such as Atlantic salmon (*Salmo salar*), but the tissue type with the highest or lowest concentration tends to vary by species (Smith *et al.,* 1999; Dalvi *et al.,* 2012).

The differences in protein concentration between broad whitefish and saffron cod has been reported in other species. Atlantic salmon, a mesothermic salmonid, showed a unique HSP expression profile relative to the eurythermal fathead minnow (*Pimephales promelas*) and mummichog (*Fundulus heteroclitus*) (Smith *et al.,* 1999). Species have unique thermal histories which, in addition to environmental factors, can impact the stress response, and these factors could have resulted in the species-specific protein concentrations observed in the current study (Iwama *et al.,* 1999). The significantly higher HSP70 protein concentration in broad whitefish supports the higher CT_max_ at the 15°C acclimation temperature relative to saffron cod. Previous research has reported higher levels of HSP70 correlates with higher acclimation temperatures, which results in an overall increase in upper thermal tolerance (Dalvi *et al.,* 2012).

### HSP70 mRNA abundance

The upregulation of HSP70a-201 and HSP70b-201 as acclimation temperature increased has been reported before in rainbow trout erythrocytes and cell lines where elevated HSP70 mRNA correlated with rising exposure temperatures (Currie *et al.,* 2000; Ojima *et al.,* 2005a; Yamashita *et al.*, 2010). The upregulated expression for 15°C-acclimated broad whitefish could explain the significantly higher CT_max_ at the same temperature as higher HSP70 transcript levels have been correlated with warmer acclimated organisms, including the longjaw mudsucker (*Gillichthys mirabilis*) (Dietz, 1994; Hofmann, 1999). Higher mRNA transcript concentration in 15°C-acclimated broad whitefish suggested that there is a temperature-dependent induction profile for HSP70 mRNA, which could explain the observed CT_max_ for this species.

The genes encoding these transcripts are paralogous and have demonstrated unique expression profiles in rainbow trout cell lines before, with HSP70b-201 having higher relative concentrations than HSP70a-201 (Ojima *et al.,* 2005b). Additionally, the heat shock transcription factor, HSF1, are encoded by two paralogous genes, which could result in unique HSP70 transcript concentrations due to differential binding rates to the heat shock element (Ojima *et al.,* 2005b). The paralogous genes encoding both the HSP70 and HSF1 transcripts could account for the unique concentrations observed in the two transcripts. Even with this differential expression, both HSP70 transcripts are vital in the heat stress response for fish, which is likely why both transcripts were significantly upregulated at the 15°C acclimation temperature (Ojima *et al.,* 2005a). Further research is needed to confirm the exact genetic mechanisms producing HSP70 transcripts in broad whitefish.

There were no significant differences in transcript concentrations between tissue types, contrasting the differences observed in HSP70 protein concentration between tissue type. The lack of tissue-specific differences could be due to differences in transcription and translation rates. Smith *et al.* (1999) found that the maximum rate of protein synthesis occurred after 2 hours of thermal shock in Atlantic salmon, but mRNA transcript concentrations continued to increase past this point. This paper suggested that existing HSP70 mRNA are translated into protein before more are transcribed. It is possible that, within the time frame of this experiment, the mRNA transcript levels had not yet increased to match the protein concentrations. There could potentially be other post-transcriptional regulations that are occurring during the heat shock response, and it is something that has been observed in Arctic charr (*Salvelinus alpinus*) and Atlantic salmon (Lewis *et al.,* 2016). Further experimentation is needed to determine what, if any, transcriptional or translational modifications are occurring in broad whitefish.

### Implications of thermal tolerance variation under ongoing climate change

There has been little to no data on the physiological and molecular parameters driving the upper thermal tolerance of broad whitefish and saffron cod. Given the accelerated rate of climate change, ecological importance of both species and the subsistence use of broad whitefish, understanding these parameters for both species will provide insight into the potential both species have in responding to elevated temperatures (Frost and Lowry, 1981; Fechhelm *et al.,* 1992; Tallman and Reist, 1997; Reusser *et al.,* 2016). The results of this study suggest that broad whitefish and saffron cod in the nearshore Beaufort Sea can shift their upper thermal tolerance due to phenotypic plasticity driven by underlying molecular mechanisms. Both species demonstrated a broad range of HSP70 protein expression, which is common for species living in a variable environment and could explain the higher CT_max_ at this temperature for 15°C-acclimated broad whitefish (White *et al.,* 1994; Currie *et al.,* 2000). Further, the higher CT_max_, acclimation response ratio and HSP70 protein concentration for broad whitefish suggests they are more physiologically capable of responding to heat stress than saffron cod. The shifts in HSP70 protein and mRNA concentrations with higher *in situ* temperature changes could result in an increased tolerance to future temperature changes (Iwama *et al.,* 1999), but it should be noted that the observed upper thermal tolerance in these lab conditions does not necessarily correlate to survivability in the nearshore Beaufort Sea if temperatures reach or exceed 15°C.

While successfully shifting an organism’s upper thermal tolerance threshold is critical to returning to organismal homeostasis, this response could also come at a cost. Studies have shown that increasing acclimation temperatures results in the convergence of the CT_max_ and incipient lethal temperatures, which limits continued acclimation (Somero, 2010). It would be beneficial to determine the upper temperature limit for broad whitefish and saffron cod to gauge their acclimation potential at temperatures warmer than 15°C. Additionally, the inducible heat shock proteins interfere with ongoing cellular processes, and the increased transcription of HSP mRNA is energetically costly and not sustainable (Feder and Hofmann, 1999; Hofmann, 1999; Lewis *et al.,* 2016). The acclimation limit and energetic expenditure of continually producing HPS70 emphasizes the potential cost of inducible thermal tolerance for these two species.

The evidence of phenotypic plasticity in broad whitefish and saffron cod suggests that they have the potential to respond to future increases in environmental temperature in the nearshore Beaufort Sea. The thermal tolerance data from this study paired with the CT_max_ measurements in other Arctic teleost species in this region shows a variety of species-specific thermal tolerance responses (Drost *et al.,* 2016; Bilyk and Sformo, 2021). Given these species-specific responses, changing thermal parameters may influence the distribution and abundance of fishes in the nearshore Beaufort Sea (Reist *et al.,* 2006; Somero, 2010; Priest *et al.,* 2022). There has already been a recent notable increase in broad whitefish and saffron cod abundance (Hamman *et al.,* 2021; Priest *et al.,* 2022). Overall, the current study provided detailed insights into the physiological and molecular parameters driving the upper thermal tolerance shifts demonstrated in broad whitefish and saffron cod, and this phenotypic plasticity may be used in response to future changes in thermal conditions in the nearshore Beaufort Sea.

## Acknowledgments

Thank you to Hilcorp Alaska LLC for providing the funding for this work and the Endicott camp for providing the infrastructure we used and logistical support. We appreciate the support of graduate students Kyle Gatt and Jonah Bacon and technicians Feyne Elmore, Sarah Barnes, Alex Page and Amanda Frantz for the assistance in fish collection and sample processing. Finally, thank you to Dr Kristen O’Brien for providing materials that were integral to completion of this project.

## Author Contributions

C.R.W. conceived the presented ideas, was the lead in all experiments, wrote the proposal to secure the URSA grant and wrote the original draft of this manuscript with input from all authors. T.M.S., A.L.K. and J.A.L. assisted in experiment conceptualization, implementation and investigation in addition to formal analysis. A.L.K. provided all materials for the HSP70 protein concentration experiment and assisted in western blot experimentation. J.A.L. wrote the proposal to secure the grant for broad whitefish RNA-seq analysis and helped submit all broad whitefish tissue samples. T.M.S. wrote the proposal to secure the Hilcorp grant that provided funding for field work in Prudhoe Bay, AK, in addition to assisting in sample collection and implementation of the infrastructure to hold all live fish samples. All authors provided critical feedback over the course of the experiment and reviewed and edited the agreed-upon final manuscript.

## Conflict of Interest

These authors declare no conflict of interest.

## Funding

This work was supported by Hillcorp Alaska, LLC [grant number 2255–00659.14.11.60], Alaska Established Program to Stimulate Competitive Research (EPSCoR) National Science Foundation (NSF) award [grant number OIA-1757348] and the Undergraduate Research and Scholarly Activity (URSA) Mentor award [grant number M21–6]. Additionally, research reported in this publication was supported by an Institutional Development Award (IDeA) from the National Institute of General Medical Sciences of the National Institutes of Health under grant number 2P20GM103395. The content is solely the authors’ responsibility and does not necessarily reflect the official views of the NIH. Funding to help with the publication of this article was provided by the University of Alaska Fairbanks Vice Chancellor for Research.

## Supplementary Material

Web_Material_coae001
