# Supplementary material for: Effects of temperature acclimation on the upper thermal tolerance of two Arctic fishes
Source: Conserv Physiol. 2024 Feb 10;12(1):coae001. doi: 10.1093/conphys/coae001 (PMC10858409; doi:10.1093/conphys/coae001)
Supplement: Web_Material_coae001 [file web_material_coae001.zip › Waterbury_Supplemental_Information.pdf]

Supplemental Information

Tables

Table S1. A concise list of all solutions and reagents used during tissue homogenization, HSP70 extraction, and Western Blot protein procedure.

**10xPBS**		**Running Buffer**	
$NaH_2PO_4H_2O$	2.03 g	Trizma Base	3.03 g
Na_2HPO_4	11.49 g	Glycine	14.4 g
NaCl	85 g	10% SDS	10 mL
	Add to 1 L dH_2O (pH 7.4)		Add to 1 L dH_2O
**Separating Gel Buffer Stock**		**Transfer Buffer**	
1.5 M Tris-HCl pH 8.8		Trizma Base	3.03 g
0.4% SDS		Glycine	14.4 g
		Methanol	20%
			Add to 1 L dH_2O
**Stacking Gel Buffer Stock**		**Blocking Buffer**	
0.5 M Trish-HCl pH 6.8		1xPBS	
0.4% SDS		5% NFDM	
**Homogenization Buffer**		**Other Necessary Reagents**	
32 mmol Tris-HCl		10% SDS	
4% SDS		29% Acrylamide : 1% Bis-acrylamide	
Add to 250 mL dH_2O (pH 6.8)		10% Ammonium persulfate	
		TEMED	
		ECL Reagent (Amersham)	
**Recipe for 10% Separating Gel**		**Recipe for Stacking Gel**	
Separating Buffer	5 mL	Separating Buffer	1.25 mL
dH_2O	8.2 mL	dH_2O	3.03 mL
29: 1% acrylamide	6.7 mL	29: 1% acrylamide	0.67 mL
10% APS	100 µL	10% APS	50 µL
TEMED	10 µL	TEMED	5 µL
**0.1% Tween**		**0.3% Tween**	
10xPBS	20 mL	10xPBS	10 mL
dH_2O	180 mL	dH_2O	90 mL
Tween	200 µL	Tween	150 µL

Table S2. The length, weight, and CT_{max} values for broad whitefish and saffron cod acclimated at 5 and 15°C.

Sample ID	Acclimation Temperature (°C)	CT_{max} Temperature (°C)	Weight (g)	Length (mm)
		broad whitefish		
BDWF_001	5	25.4	12.65	112
BDWF_003	5	25.9	8.60	93
BDWF_004	5	24.8	9.23	110
BDWF_005	5	21.2	11.67	111
BDWF_006	5	23.7	26.94	147
BDWF_007	5	24.8	10.52	110
BDWF_008	5	23.5	14.50	117
BDWF_009	5	22.4	10.70	112
BDWF_002	15	23.7	10.36	107
BDWF_010	15	26.2	13.80	117
BDWF_011	15	28.6	11.79	114
BDWF_012	15	27.5	31.97	148
BDWF_013	15	27.1	12.20	110
BDWF_014	15	27.7	8.59	102
BDWF_015	15	26.0	11.20	104
BDWF_016	15	28.8	12.02	111
BDWF_017	15	27.6	11.01	112
		saffron cod		
SFCD_001	15	24.9	15.05	152
SFCD_002	15	25.3	14.27	143
SFCD_003	15	25.3	11.87	140
SFCD_004	15	25.4	17.23	149
SFCD_005	15	26.1	12.81	131
SFCD_006	15	26.1	8.00	121
SFCD_007	15	26.6	14.40	130
SFCD_009	15	21.9	18.23	157
SFCD_010	15	25.5	12.16	133
SFCD_011	15	25.7	22.40	163
SFCD_012	15	25.6	17.39	152
SFCD_013	15	25.8	12.62	129
SFCD_014	15	26.8	19.20	157
SFCD_015	15	26.7	15.67	146
SFCD_016	15	27.1	9.84	130
SFCD_017	5	22.0	20.82	156
SFCD_018	5	22.1	19.47	153
SFCD_019	5	22.1	19.07	152
SFCD_020	5	23.8	23.02	159
SFCD_021	5	23.8	NA	159
SFCD_022	5	23.7	19.39	155
SFCD_023	5	23.8	15.70	149
SFCD_024	5	24.3	12.21	130
SFCD_025	5	22.0	25.26	164
SFCD_026	5	22.6	21.06	130
SFCD_027	5	23.3	26.09	170
SFCD_028	5	23.2	18.65	145
SFCD_029	5	23.8	15.82	149
SFCD_030	5	23.7	24.14	160

SFCD_031	5	23.6	16.49	146
SFCD_032	5	23.7	20.21	151

Table S3. The statistical parameters resulting from the Wilcoxon rank-sum test from comparing the CT_{max} values between each acclimation temperature in addition to between broad whitefish and saffron cod. All reported p-values are Bonferroni corrected. Any significant values (α = 0.05) are bolded.

Comparative Groups	Test Group	N_1	N_2	Statistic	P-Value
15°C vs 5°C	broad whitefish	9	8	72	**6.26×10^{-4}**
	saffron cod	14	16	224	**3.48×10^{-6}**
broad whitefish vs. saffron cod	15°C	9	14	110	**6.78×10^{-3}**
	5°C	8	16	81	6.22×10^{-1}

Table S4. Statistical parameters resulting from the Wilcoxon rank-sum test from comparing the HSP70 concentration between acclimation temperature and species. All reported p-values are Bonferroni corrected. Any significant values (α = 0.05) are bolded.

Comparative Group	Tissue	N_1	N_2	T-Statistic	P-Value
		broad whitefish			
15°C vs 5°C	Liver	9	8	12.5	0.1608
	Muscle	9	8	16.0	0.3552
	Brain	9	8	17.0	0.4470
		saffron cod			
15°C vs 5°C	Liver	7	14	8.5	**1.7×10^{-2}**
	Muscle	15	16	118.5	1.0000
	Brain	15	16	148.0	1.0000
		Acclimation Temperature: 15°C			
broad whitefish vs. saffron cod	Liver	9	7	63	**1.05×10^{-3}**
	Muscle	9	15	133	**6.36×10^{-4}**
	Brain	9	15	44	1.0000
		Acclimation Temperature: 5°C			
broad whitefish vs. saffron cod	Liver	8	14	112	**3.75×10^{-5}**
	Muscle	8	16	128	**6.00×10^{-4}**
	Brain	8	16	76	1.0000

Table S5. The Kruskal-Wallis statistical parameters for the HSP70 concentration between muscle, liver, and cranial tissue samples at the two acclimation temperatures for broad whitefish and saffron cod. A Dunn's post-hoc was performed if the p-value was significant, and these post-hoc values with a Bonferroni correction are italicized. Any significant statistical parameters are indicated by bolded values (α = 0.05).

Comparative Group	χ^2	DF	p-value
broad whitefish (5°C)	14.615	2	**6.70×10^{-4}**
*CT*HT*			*4.719×10^{-1}*
*CT*MT*			***4.648×10^{-4}***
*MT*HT*			*5.353×10^{-2}*
broad whitefish (15°C)	20.956	2	**2.82×10^{-5}**
*CT*HT*			***4.113×10^{-2}***
*CT*MT*			***1.441×10^{-5}***
*MT*HT*			*1.050×10^{-1}*
saffron cod (5°C)	38.5	2	**4.47×10^{-9}**
*CT*HT*			***4.24×10^{-3}***
*CT*MT*			***1.73×10^{-9}***
*MT*HT*			***1.56×10^{-2}***
saffron cod (15°C)	26.9	2	**1.42×10^{-6}**
*CT*HT*			***5.81×10^{-3}***
*CT*MT*			***1.31×10^{-6}***
*MT*HT*			*1.000*

Table S6. Statistical parameters resulting from the Wilcoxon rank-sum test comparing the mRNA expression between liver and muscle samples in broad whitefish at each acclimation temperature. All reported p-values are Bonferroni corrected and any significant values ($\alpha = 0.05$) are bolded.

	**Comparative group**	**Temperature Treatment**	**N_1**	**N_2**	**T-Statistic**	**P-Value**
Transcript A	Liver vs. Muscle	15°C	6	6	9.00	0.54
		5°C	6	5	13.00	1.00
		Control (8°C)	4	4	2.00	0.342
Transcript B	Liver vs. Muscle	15°C	6	6	9.00	0.54
		5°C	6	5	13.00	1.00
		Control (8°C)	4	4	0.00	0.0858

Table S7. Kruskal-Wallis statistical parameters for the mRNA expression in the broad whitefish muscle and liver tissue samples. A Dunn's post-hoc test was performed if the p-value was significant, and these post-hoc values with a Bonferroni correction are italicized. Any significant statistical parameters are indicated by bolded values ($\alpha = 0.05$).

**Comparative Group**	**χ^2**	**DF**	**p-value**
Liver Tissue – HSP70A	9.94	2	**6.94×10^{-3}**
*15°C *5°C*			*6.76×10^{-1}*
*15°C *Control (8°C)*			***4.97×10^{-3}***
*5°C *Control (8°C)*			*1.18×10^{-1}*
Muscle Tissue – HSP70A	11.2	2	**3.65×10^{-3}**
*15°C *5°C*			*7.29×10^{-2}*
*15°C *Control (8°C)*			***4.06×10^{-3}***
*5°C *Control (8°C)*			*8.81×10^{-1}*
Liver Tissue – HSP70B	9.94	2	**6.94×10^{-3}**
*15°C *5°C*			*6.76×10^{-1}*
*15°C *Control (8°C)*			***4.97×10^{-3}***
*5°C *Control (8°C)*			*1.18×10^{-1}*
Muscle Tissue – HSP70B	10.7	2	**4.78×10^{-3}**
*15°C *5°C*			***4.91×10^{-2}***
*15°C *Control (8°C)*			***7.31×10^{-3}***
*5°C *Control (8°C)*			*1.00*

Figures

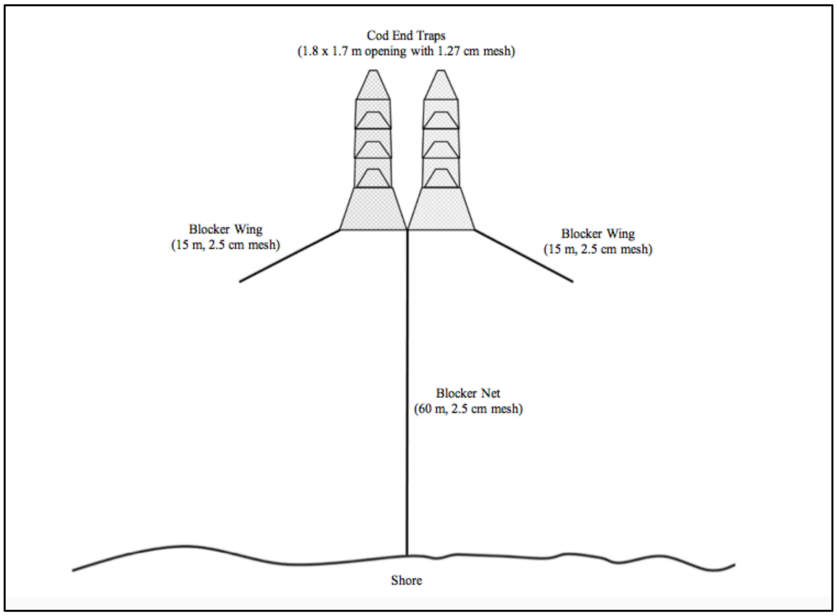

Figure S1. Overhead view of the nets used at each site during sampling for broad whitefish and saffron cod (Priest *et al.,* 2018).

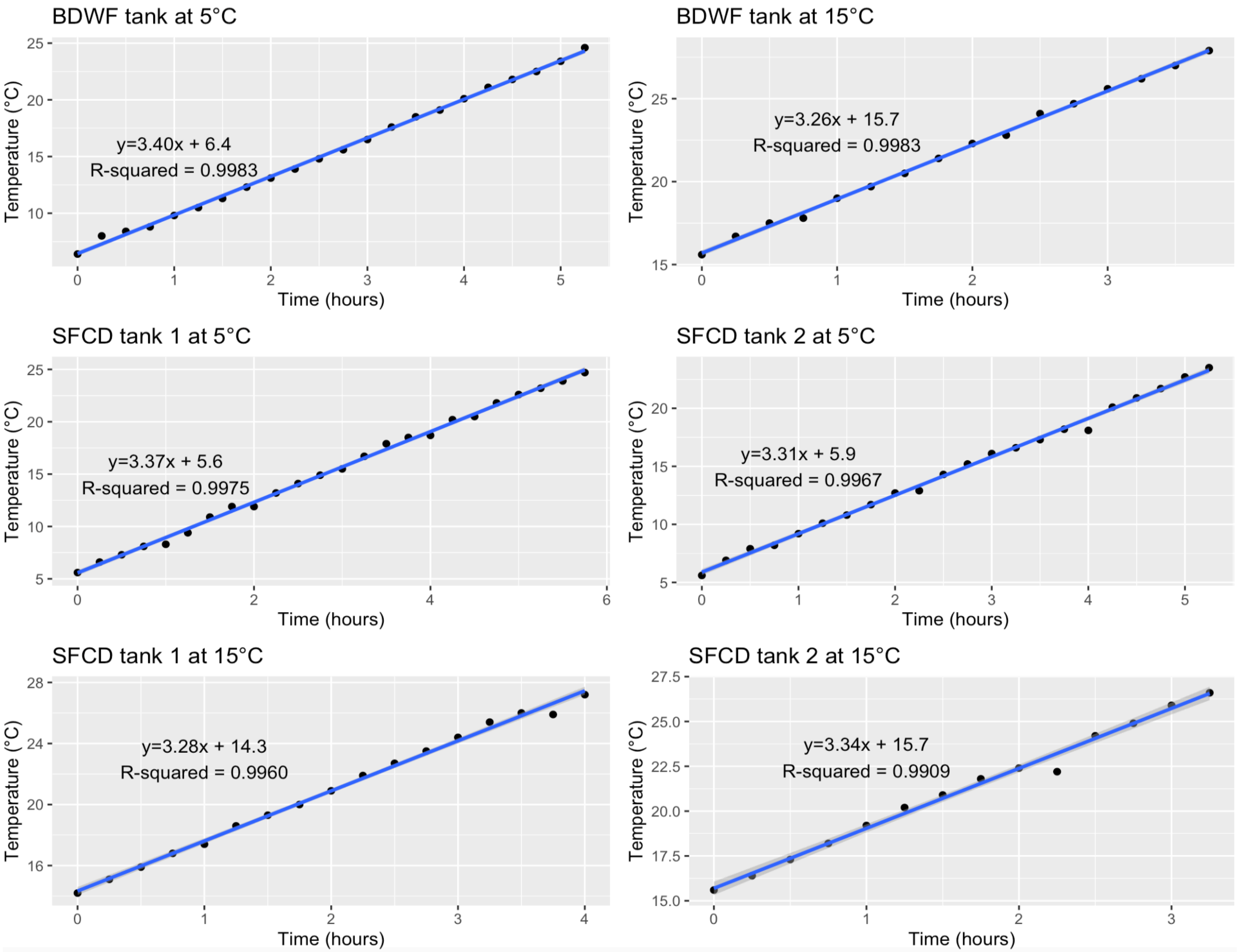

Figure S2. The increase in temperature over the course of the CT_{max} experiment for each experimental tank.

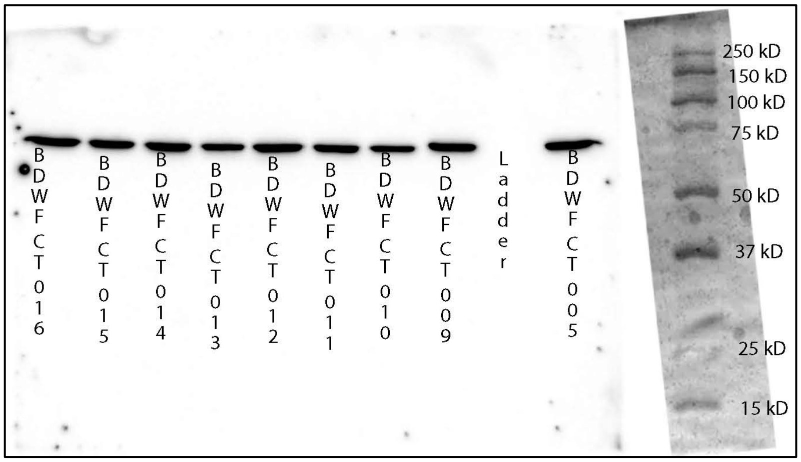

Figure S3. A sample of an imaged membrane that would be later used in densitometric analysis. This membrane contains samples from broad whitefish (BDWF) cranial tissue (CT) samples 009-016 with the internal standard and protein ladder. The ladder reference on the right was imaged using the colorimetric marker setting. Otherwise, the membranes were imaged without this setting, which is why there appears to be an empty well marked by ladder. The overlay of the ladder with the sample membrane was to confirm that the proteins separated and targeted were 70 kDa.

Supplementary procedure 1

Wet tank transfer Western Blot Protocol

A 10% SDS gel was created by first pouring a 10% separating gel in a glass plate with 1 mm spacers, letting it set (~ 1 hour), and then pouring the stacking gel fitted with a 10-well comb and letting that polymerize (~45 minutes, Table S1). Once the gels had polymerized, the samples were prepared by diluting each sample with dH_2O so that the final concentration was 1.5 $\mu g\ \mu L^{-1}$. Next, 2 x Laemmli Sample Buffer (Bio-Rad, Hercules, California) was mixed 20:1 with ß-mercaptoethanol (Bio-Rad, Hercules, California) before adding 10 µL to each sample. Eight samples were prepared per gel in addition to the Precision Plus Protein™ Kaleidoscope™ Prestained Protein Standard (Bio-Rad, Hercules, California) and the internal standard, which was kept the same in every Western Blot. The internal standard used was the broad whitefish sample BDWF005_CT because it showed high, consistent specificity with the HSP70 antibodies. The Prestained Protein Standard was diluted 1:1 with diH_2O to make 10 µL before being mixed with 10 µL of the sample buffer. Every sample minus the protein standard was denatured in a heating block at 100°C for 3 minutes before being allowed to cool to room temperature. Finally, all samples were centrifuged at 15,000 RPM for 30 seconds.

The gels were assembled in the Mini-PROTEAN Tetra Cell (Bio-Rad, Hercules, California) followed by the addition of running buffer (Table S1). The samples were then loaded in to each well and run at 150 V until the dye front ran out of the bottom of the gel (~ 1 hour). The following components were soaked in transfer buffer (Table S1) before being assembled as described. A piece of nitrocellulose membrane (0.45 µm, GE Healthcare, Chicago, Illinois) was fitted over the gel before both were sandwiched between cardboard and sponge pads. The entire

ensemble was placed in the Tetra Blotting Module (Bio-Rad, Hercules, California), the tank filled with transfer buffer, and the transfer allowed to occur overnight at 30 V.

Once transfer was complete, the membrane was immediately blocked with blocking buffer (Supplementary table 1) for an hour on a rotating table. After an hour the buffer was discarded and the membranes were allowed to intubate with a monoclonal mouse anti-HSP70 antibody (MA3-006, ThermoFisher Scientific, Waltham, Massachusetts) at 1:1,000 dilution with blocking buffer for an hour and a half. The primary antibody was saved for future use, and the membranes were rinsed 3 x 10 minutes with 0.1% Tween (Table S1). The solution was then poured out and the membranes were mixed with Donkey anti-Mouse IgG (H+L) Secondary Antibody, HRP (ThermoFisher Scientific, Waltham, Massachusetts) at 1:10,000 dilution with blocking buffer for an hour. The secondary antibody was discarded, and the membrane rinsed 3 x 5 minutes with 0.3% Tween followed by 3 x 5 minutes with 0.1% Tween (Table S1). The membranes were prepared for imaging by adding SuperSignal West Pico PLUS Chemiluminescent Substrate (ThermoFisher Scientific, Waltham, Massachusetts) and letting the membranes sit for five minutes. Finally, the membranes were imaged with the Amersham Imager 600 (GE Healthcare, Chicago, Illinois) using the chemiluminescent setting with exposure times of 30 seconds, 1 minute, 5 minutes, and 10 minutes (if needed).
